# Genome‐wide identification of tolerance mechanisms toward *p*‐coumaric acid in *Pseudomonas putida*

**DOI:** 10.1002/bit.26495

**Published:** 2017-11-28

**Authors:** Patricia Calero, Sheila I. Jensen, Klara Bojanovič, Rebecca M. Lennen, Anna Koza, Alex T. Nielsen

**Affiliations:** ^1^ Novo Nordisk Foundation Center for Biosustainability Technical University of Denmark Kemitorvet Lyngby Denmark

**Keywords:** *p*‐coumaric acid, *Pseudomonas putida*, Tn‐seq, tolerance

## Abstract

The soil bacterium *Pseudomonas putida* KT2440 has gained increasing biotechnological interest due to its ability to tolerate different types of stress. Here, the tolerance of *P. putida* KT2440 toward eleven toxic chemical compounds was investigated. *P. putida* was found to be significantly more tolerant toward three of the eleven compounds when compared to *Escherichia coli*. Increased tolerance was for example found toward *p*‐coumaric acid, an interesting precursor for polymerization with a significant industrial relevance. The tolerance mechanism was therefore investigated using the genome‐wide approach, Tn‐seq. Libraries containing a large number of miniTn5‐Km transposon insertion mutants were grown in the presence and absence of *p‐*coumaric acid, and the enrichment or depletion of mutants was quantified by high‐throughput sequencing. Several genes, including the ABC transporter Ttg2ABC and the cytochrome *c* maturation system (*ccm*), were identified to play an important role in the tolerance toward *p*‐coumaric acid of this bacterium. Most of the identified genes were involved in membrane stability, suggesting that tolerance toward *p*‐coumaric acid is related to transport and membrane integrity.

## 1 INTRODUCTION

*Pseudomonas putida* is one of the soil organisms that is gaining interest due to its natural resistance to a number of hydrophobic solvents, such as xylenes, toluene, or styrene (Cruden, Wolfram, Rogers, & Gibson, 1992; Inoue, Yamamoto, & Horikoshi, 1991; Weber, Ooijkaas, Schemen, Hartmans, & De Bont, 1993). Some of the mechanisms leading to increased tolerance in these solvent‐tolerant strains have been identified and described, and efflux pumps (Kieboom & de Bont, 2001; Mosqueda & Ramos, 2000; Ramos, Duque, Godoy, & Segura, 1998) as well as changes in the membrane composition (Heipieper & De Bont, 1994), have been shown to be important for tolerance in these strains. Some of the changes that can occur in the membrane, when bacteria are exposed to hydrophobic solvents, include vesicle formation, alteration of phospholipid composition, and reduced permeability of the cell membrane (Heipieper & De Bont, 1994; Nicolaou, Gaida, & Papoutsakis, 2010; Ramos et al., 2002). The mechanisms behind tolerance in the model strain *P. putida* KT2440 has also been studied for different compounds (Benndorf, Thiersch, Loffhagen, Kunath, & Harms, 2006; Domínguez‐Cuevas, González‐Pastor, Marqués, Ramos, & de Lorenzo, 2006; Fernandez, Conde et al., 2012; Fernandez, Niqui‐Arroyo, Conde, Ramos, & Duque, 2012; Roca, Rodríguez‐Herva, Duque, & Ramos, 2008; Santos, Benndorf, & Sá‐Correia, 2004).

**Figure 1:**
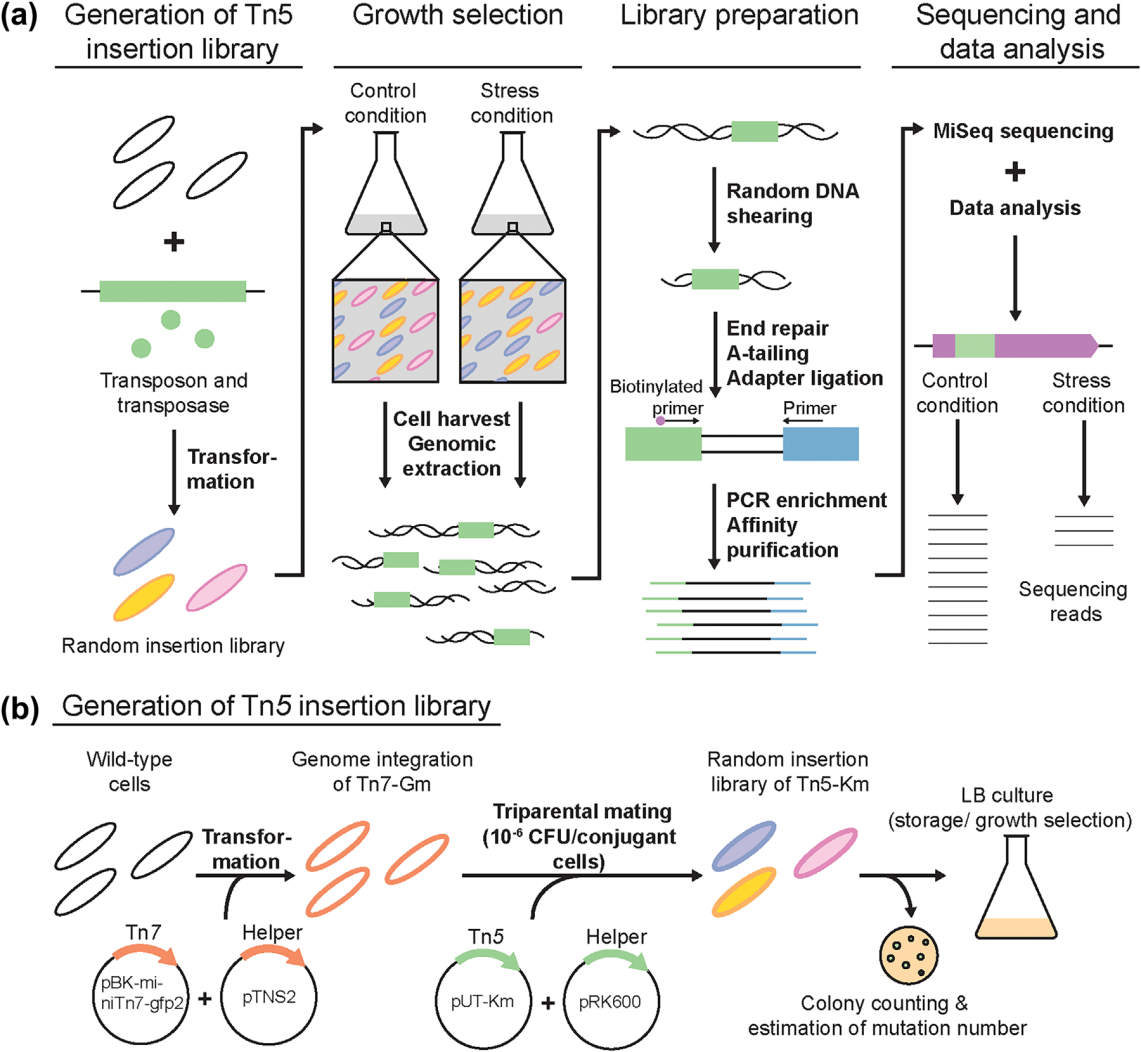
(a) Diagram of the experimental procedure used in a Tn‐seq approach. A Tn*5* insertion library (transposons depicted as green boxes) is constructed by transformation and used in growth assays in different conditions, in order to assess differential growth of each insertion mutant. After harvesting the cells, the extracted genomic DNA is processed by DNA shearing, A‐tailing, and ligation of adapters (blue boxes), followed by an enrichment using PCR. This enriched library is subsequently sequenced by NGS and the results of the number of readings in the different conditions are compared in order to identify the differential abundance of insertions. (b) The method used in this study for generating the Tn*5* library generating a combination of miniTn*7*‐Gm and miniTn*5*‐Km. The efficiency of the method to generate random insertion libraries was assessed by counting colonies of a sample

Traditionally, transposon insertion mutagenesis has been used to identify gene functions in many different bacteria (Glass et al., 2006; Hensel et al., 1995; Jacobs et al., 2003; Mack et al., 1994), including *P. putida* KT2440 (de Lorenzo & Timmis, 1994; Herrero, Lorenzo, & Timmis, 1990; Molina‐Henares et al., 2010). However, genome wide screening and identification of all genes involved in tolerance has been a limiting factor, which in the last years have been overcome through the use of next generation sequencing (NGS) techniques. Transposon insertion sequencing (Tn‐seq) (Figure 1a), enables high‐throughput identification of genomic regions involved in the survival of cells when exposed to various conditions (Barquist, Boinett, & Cain, 2013; van Opijnen & Camilli, 2013). A number of organisms have been investigated using this technique, thereby successfully identifying genes important for growth under different stress conditions, essentially by simultaneously investigating the fitness of all single mutants (Gawronski, Wong, Giannoukos, Ward, & Akerley, 2009; Langridge et al., 2009; Lennen & Herrgård, 2014; Santiago et al., 2015). So far, a Tn‐seq method has, however, not been developed for *P. putida*.

Metabolic engineering of microorganisms for production of chemical compounds from renewable resources aims at providing an alternative to petroleum‐based chemical processes. However, it still faces some challenges, such as ameliorating the toxic effects of intermediates and products of interest (Keasling, 2010). Numerous studies have focused on increasing the tolerance of different production strains toward various biochemicals using different strategies (Alper, Moxley, Nevoigt, Fink, & Stephanopoulos, 2006; Atsumi et al., 2010; Dunlop et al., 2011; Goodarzi et al., 2010). An alternative to this approach is to identify alternative production hosts that are naturally tolerant to higher concentrations of the chemicals of interest. *P. putida* is one such strain that is often utilized for bioremediation and production of more toxic chemicals (Loeschcke & Thies, 2015; Nikel, Martínez‐García, & de Lorenzo, 2014; Poblete‐Castro, Becker, Dohnt, dos Santos, & Wittmann, 2012; Verhoef, Ruijssenaars, de Bont, & Wery, 2007; Verhoef, Wierckx, Westerhof, De Winde, & Ruijssenaars, 2009).

In this study, we have investigated the tolerance of *P. putida* KT2440 toward a range of different biochemicals and found that it exhibits significantly increased tolerance toward the aromatic organic compound *p*‐coumaric acid, a low molecular weight phenolic acid present in soils and plants (Mussatto, Dragone, & Roberto, 2007; Strobel, 2001). *p*‐Coumaric acid is released as an inhibitory by‐product during the pretreatment of the lignocellulosic feedstocks (Jönsson & Martín, 2016). There is furthermore significant industrial interest in producing *p*‐coumaric acid since it can be used for making high performance polymers. Aditionally, it is a precursor for a number of interesting high value compounds (Santos, Koffas, & Stephanopoulos, 2011).

To study the mechanism of tolerance toward *p*‐coumaric acid, we developed a novel method for generating random transposon insertion mutant libraries in *P. putida* KT2440. In combination with transposon insertion sequencing, the most important genes required for growth in the presence of *p*‐coumaric acid were identified. A number of genes involved in maintenance of the membrane structure and efflux of solvent compounds were found to be important for growth, when *P. putida* KT2440 was exposed to this compound.

## 2 EXPERIMENTAL PROCEDURES

### 2.1 Bacterial strains and chemicals

*P. putida* KT2440 (DSM‐6125) and *P. putida* KT2440 Δ*fcs* (Calero, Jensen, & Nielsen, 2016) were grown at 30°C and *E. coli* K‐12 MG1655 was grown at 37°C. Cells were routinely cultured in LB medium or on LB agar plates according to standard protocols (Sambrook & Russel, 2000). Modified M9 minimal medium containing 5 g L^−1^ of glucose as carbon source (Abril, Michan, Timmis, & Ramos, 1989) was used for all assays. *E. coli* DH5α and *E. coli* DH5α‐Pir were used for plasmid maintenance and were grown in LB medium at 37°C.

Antibiotics and other supplements were used at the following concentrations: chloramphenicol (Cm) 30 µg ml^−1^, kanamycin (Km) 50 µg ml^−1^, and gentamicin (Gm) 10 µg ml^−1^. A total of 300 mM sucrose was used for preparing electrocompetent *P. putida* cells. The chemicals used for toxicity screening included sodium acetate (Sigma, S8750), *n*‐butanol (Sigma, 281549), 3‐hydroxy‐γ‐butyrolactone (TCI Chemicals, H0939), 1,4‐butanediol (Merck, 801534), furfural (Sigma, 185914), itaconic acid (Sigma, I29204), levulinic acid (Sigma, L2009), succinic acid (Sigma, S9512), L‐threonine (Sigma, T8441), *p*‐coumaric acid (TCI Chemicals, C0393), and octanoic acid (Sigma, O3907). Stocks of these chemicals were prepared in modified minimal medium, and neutralized with NaOH to pH 7 when acidic chemicals were used.

### 2.2 Plasmids and strains constructions

*P. putida* KT2440::miniTn*7*‐Gm:GFP and *P. putida* KT2440 Δ*fcs*::miniTn*7*‐Gm:GFP (Table 1) were constructed by transformation of plasmid pBK‐miniTn7‐gfp2 (Koch, Jensen, & Nybroe, 2001) as previously described (Choi, Kumar, & Schweizer, 2006), using plasmid pTNS2 (Choi et al., 2005) as a helper. The correct integration of the transposon was checked by colony PCR using primers Tn7‐GlmS and Tn7R109 (Table 2).

**Table 1:** Plasmids and strains used in this study

	Genotype	Source
Plasmid		
pBK‐miniTn7‐gfp2	pUC19‐based delivery plasmid for miniTn7‐gfp2. GmR, CmR, ApR, mobq	Koch et al. (2001)
pTNS2	R6K replicon‐based helper plasmid, providing the Tn7 transposition functions in trans. ApR Mob+	Choi et al. (2005)
pUT‐Km	R6K replication origin‐based suicide delivery plasmid for miniTn5‐Km. ApR KmR	Herrero et al. (1990)
pRK600	ColE1 *oriV*; RP4tra+ RP4oriT; CmR; helper in triparental matings	Kessler, de Lorenzo, and Timmis (1992)
pEMG	*oriV*(R6K), lacZα fragment with I‐SceI sites; KmR	Martínez‐García and de Lorenzo (2011)
pEMG‐vacJ	*oriV*(R6K), lacZα fragment with I‐SceI sites, with 500 bp of gene *vacJ*; KmR	This study
pEMG‐tyrB	*oriV*(R6K), *lacZα* fragment with I‐*Sce*I sites, with 500 bp of gene *tyrB*; KmR	This study
pEMG‐fleN	*oriV*(R6K), *lacZα* fragment with I‐*Sce*I sites, with 500 bp of gene *fleN*; KmR	This study
Strains		
*P*. *putida* KT2440	Mt‐2 *hsdR*1 (r^‐^ m^+^)	Bagdasarian et al. (1981)
*P*. *putida* KT2440 Δ*fcs*	*P. putida* KT2440 derivative with gene *fcs* deleted	Calero et al. (2016)
*P. putida* KT2440::miniTn7‐Gm:GFP	*P. putida* KT2440 with Gentamycin resistance gene in a miniTn7	This study
*P. putida* KT2440 Δ*fcs*::miniTn7‐Gm:GFP	*P. putida* KT2440 *Δfcs* with Gentamycin resistance gene in a miniTn7	This study
*P*. *putida* KT2440 *ttg2A^‐^*	*P. putida* KT2440 derivative with a miniTn5‐Km insertion gene *ttg2A*	Duque et al. (2007)
*P. putida* KT2440 *ttg2B^‐^*	*P. putida* KT2440 derivative with a miniTn5‐Km insertion gene *ttg2B*	Duque et al. (2007)
*P. putida* KT2440 *ccmC^‐^*	*P. putida* KT2440 derivative with a miniTn5‐Km insertion gene *ccmC*	Duque et al. (2007)
*P. putida* KT2440 *ccmF^‐^*	*P. putida* KT2440 derivative with a miniTn5‐Km insertion gene *ccmF*	Duque et al. (2007)
*P. putida* KT2440 *vacJ^‐^*	*P. putida* KT2440 derivative with an insertion in gene *vacJ*	This study Duque et al. (2007)
*P. putida* KT2440 *tyrB^‐^*	*P. putida* KT2440 derivative with an insertion in gene *tyrB*	This study Duque et al. (2007)
*P. putida* KT2440 *fleN^‐^*	*P. putida* KT2440 derivative with an insertion in gene *fleN*	This study Duque et al. (2007)
*E. coli* K‐12 MG1655	F^‐^ *hsdS gal*	
*E. coli* DH5α	ϕ80d*lac*ZΔM15 Δ(*lacZYA‐argF*)U169 *recA1 endA*1 *hsdR17 (rk* ^‐^ mk^+^) *sup*E44 *thi*‐1 gyrA relA1	Hanahan (1985)

**Table 2:** Oligonucleotides used in this study

Oligonucleotide	Sequence	Purpose
Tn7‐GlmS	AATCTGGCCAAGTCGGTGAC	Check miniTn*7* integration after *glmS*
Tn7R109	CAGCATAACTGGACTGATTTCAG	
BioTEG‐Tn5‐fw	AATGATACGGCGACCACCGAGATCTACACTCTTTCCCTACACGACGCTCTTCCGATCT**AGCCGGATCCTCTAGAGTCGACC**	Biotinylated primer with TEG in 5′ for enrichment of the Tn*5* fragments. Bold sequence corresponds to the sequence that anneals with the transposon.
Tn5seq_rev	CAAGCAGAAGACGGCATACGAGAT	Enrichment of the Tn*5* fragments.
VacJ‐ins‐U‐fw	*ATCTGAGTU* **TAATTAATTAATTA**GGGAAGCGGTCAACCGCCC	USER‐cloning of the homologous region of gene *vacJ* in pEMG. In bold STOP codons. In italics overhangs homologous to pEMG_phuser_rv.
vacJ‐ins‐U‐rv	*ATCCCTAGAAAU* **TAATTAATTAATTA**CTTTTCGGCCGAGAGCAGGCTG	Same as the previous one. In italics overhangs homologous to pEMG_phuser_fw.
TyrB‐ins‐U‐fw	*ATCTGAGTU* **TAATTAATTAATTA**GATACCATCTGCTTGCGCATG	Same as the previous pair fot gene *tyrB*.
TyrB‐ins‐U‐rv	*ATCCCTAGAAAU* **TAATTAATTAATTA**GGCATGCTCGAAGACCTCAAC	Same as the previous pair fot gene *tyrB*.
FleN‐ins‐U‐fw	*ATCTGAGTU* **TAATTAATTAATTA**TCGTAGACGGCACGCTGCTTC	Same as the previous pair fot gene *fleN*.
FleN‐ins‐U‐rv	*ATCCCTAGAAAU* **TAATTAATTAATTA**CTCGCCGATGTGATTGAAGGGC	Same as the previous pair fot gene *fleN*.
pEMG_phuser_fw	ATTTCTAGGGAUAACAGGGTAATCCGGCGTAATCAT	Cloning in pEMG
pEMG_phuser_rv	AACTCAGAUTACCCTGTTATCCCTATACTGGCC	Cloning in pEMG

Mutants in genes *ttg2A*, *ttg2B*, *ccmC*, and *ccmF* were obtained from the Pseudomonas Reference Culture Collection (PRCC), consisting of insertion mutants generated by disruption of the genes using a miniTn5‐Km (Duque et al., 2007).

Mutants in genes *vacJ*, *tyrB*, and *fleN* were constructed by introducing, by homologous recombination, a 3 kb fragment of DNA with a kanamycin resistance gene for its selection. For this purpose, plasmids pEMG‐vacJ, pEMG‐tyrB and pEMG‐fleN were constructed by cloning a 500 bp homologous fragment of the gene to be disrupted, using USER‐cloning (Nour‐Eldin, Geu‐Flores, & Halkier, 2010). The correct plasmids, which were checked by colony PCR and sequencing, were electroporated into *P. putida* KT2440 strains and the resistance to kanamycin was selected. The correct insertion was checked by PCR.

### 2.3 Chemical tolerance assays

Overnight cultures of *P. putida* KT2440 were diluted 20‐fold in 10 ml of modified M9 minimal medium. Cells were grown in 96‐well microtiter plates (flat bottom, Greiner Bio‐one, Frickenhausen, Germany) at 30°C and 200 rpm in an ELx808 microtiter plate reader (BioTek, Winooski, VT). After 3 hr of incubation, the cultures had reached an OD_630_ of approximately 0.1, and the stressors were added at different concentrations. Cells exposed to the different chemicals at various concentrations were incubated in a microtiter plate reader for 24 hr, and growth was followed by measuring OD_630_ every 30 min. The concentrations used for each compound were: 0, 2.25, 9, 22.5, and 45 g L^−1^ of threonine; 0, 8, 20, 30, and 40 g L^−1^ of itaconic acid; 0, 7.3, 18.18, 27.27, and 36.36 g L^−1^ of succinic acid; 0, 0.25, 0.5, 2.5, and 5 % (v/v) of 3‐hydroxy‐butyrolactone; 0, 5, 10, 50, and 100 g L^−1^ of sodium acetate; 0, 0.1, 0.5, 2.5, and 5 % (v/v) of levulinic acid; 0, 0.075, 0.15, 0.75, and 1.5 % (v/v) of furfural; 0, 0.25, 0.5, 2.5, and 5 % (v/v) of 1,4‐butanediol; 0, 5, 10, 50, and 100 g L^−1^ of butanol; and 0, 15, 30, and 60 mM of *p*‐coumaric acid.

Due to absorbance interference of the compound in the readings in 96‐well microtiter plates, octanoic acid toxicity assays in *P. putida* KT2440 and *E. coli* K‐12 MG1655 were performed in 50 ml of modified M9 minimal medium in 250 ml shake flasks at 30 and 37°C, respectively, and 250 rpm of shaking. Overnight cultures grown in modified M9 minimal medium were diluted to obtain an initial OD_600_ of 0.1 and incubated until OD_600_ reached 0.5, after which octanoic acid was added to the culture. The concentrations used were 0, 30, 40, 60, and 100 mM. Growth was followed by measuring OD_600_ every hour for 5 hr.

*p*‐Coumaric acid tolerance in strains *P. putida* KT2440 and *P. putida* KT2440 Δ*fcs, ttg2A^‐^, ttg2B^‐^, ccmC^‐^, ccmF^‐^, vacJ^‐^, tyrB^‐^, or fleN^‐^*, as well as in *E. coli* K‐12 MG1655 was tested following the same protocol described above using 0, 15, and 30 mM of *p*‐coumaric acid.

### 2.4 Generation of miniTn*5* insertions libraries

Introduction of plasmid pUT‐Km into *P. putida* KT2440::miniTn*7*‐Gm and *P. putida* KT2440 Δ*fcs*::miniTn*7*‐Gm was performed by triparental mating. A total of 20 ml of overnight LB pre‐cultures of the recipient strains, the donor strain containing pUT‐Km, and the strain containing the helper plasmid pRK600, were harvested and washed twice in a phosphate buffer, after which they were mixed in a 1:1:1 ratio and adjusted to a volume of 1 ml in phosphate buffer. A volume of 100 µl was placed on an autoclaved 0.45 µm filter (Durapore membrane filters, Merck Millipore, Hellerup, Denmark), using a total of 10 filters, and incubated overnight at 30°C on LB plates. Subsequently, cells on all filters were re‐suspended in 8 ml of buffer. A total of 100 µl of this mix was diluted and plated on LB agar plates containing kanamycin and gentamycin for counting the number of mutants in the library, and the rest was used to inoculate 400 ml of LB with kanamycin and gentamycin in a 2 L shake flask, which was incubated at 30°C with 250 rpm shaking overnight. Aliquots of this culture were used to prepare cryo stocks in glycerol and stored at −80°C.

### 2.5 Transposon mutant library growth selection

Tn*5* mutant libraries of *P. putida* KT2440::miniTn*7*‐Gm and *P. putida* KT2440 Δ*fcs*::miniTn*7*‐Gm were used for growth selection under *p*‐coumaric acid stress conditions. The cryo stocks, containing approximately 100,000 mutants, determined by colony counting on agar plates, were thawed on ice, washed twice with LB and grown overnight in 50 ml of LB with proper antibiotics at 30°C in 250 ml shake flasks. A total of 10 ml of the overnight LB culture was harvested for genomic DNA extraction. Part of the overnight LB culture was used to inoculate 50 ml of modified M9 minimal medium with antibiotics to a starting OD_600_ of 0.1 after washing the cells with modified M9 minimal medium. Cells were incubated at 30°C and 250 rpm and growth was followed by measuring OD_600_ every 2 hr. When cultures reached an OD_600_ of 0.5, they were divided into 25 ml cultures and diluted two fold in preheated minimal medium or in minimal medium with 50 mM of *p*‐coumaric acid. Growth was followed until cultures reached late exponential phase at an OD_600_ of approximately 2, after which the cells were harvested and stored at −20°C prior to gDNA extraction. Three independent biological replicates were carried out.

### 2.6 Library preparation for Tn‐seq

gDNA libraries were prepared as previously described (Lennen & Herrgård, 2014). *P. putida* gDNA was extracted using the PureLink genomic DNA kit (Invitrogen, Thermo Fisher, Waltham, MA) and 3 µg of total gDNA was sheared in 300‐bp fragment size using a Covaris E220 ultrasonicator. End repair (NEBNext End repair module, New England Biolabs, Ipswich, MA) and A‐tailing using Klenow Fragment (Thermo Scientific, Waltham, MA) was performed on the fragments prior to ligation of adapters. Illumina TruSeq adapters were ligated using the NEBNext Quick Ligation Module (New England Biolab) and the transposon‐chromosome junctions were amplified by PCR with a primer specific for the adapter and a biotinylated primer specific for the miniTn*5* inserts. Biotinylated PCR products were subsequently purified by affinity capture using the Dynal MyOne Streptavidin C1 beads (Invitrogen). Library size distribution was validated by qPCR, using the KAPA qPCR quantification kit, the Agilent RNA 6000 Pico kit, and the Agilent 2100 Bioanalyzer. Finally, the library was sequenced on an Illumina MiSeq.

### 2.7 Data analysis

Reads obtained from sequencing were initially processed in order to discard the unspecific sequences and trim the length of the correct ones to 120 bp, leaving 25 bp of genomic sequence that can be used for comparison to the *P. putida* KT2440 genome. Genes that were conditionally more abundant were identified using the ESSENTIALS pipeline (Zomer, Burghout, Bootsma, Hermans, & van Hijum, 2012). Gene‐level insertion counts were normalized using TMM normalization and *p*‐values adjusted by Benjamini‐Hochberg correction. Genbank version NC_002947.3 of *P. putida* KT2440 was used as the reference genome.

## 3 RESULTS

### 3.1 Tolerance of *P. putida* KT2440 toward industrially relevant biochemicals

*P. putida* has been widely used for bioremediation because of its high degree of tolerance toward many organic compounds, and it is furthermore a promising host for metabolic engineering. We therefore decided to screen *P. putida* KT2440 for its tolerance toward a number of chemical compounds that could be relevant for biobased production, and that are known to be relatively toxic to *E. coli* (Rau, Calero, Lennen, Long, & Nielsen, 2016). Six of the compounds, hydroxy‐*γ*‐butyrolactone, furfural, itaconic acid, levulinic acid, succinic acid, and L‐threonine, have previously been identified as being among the top 30 predicted chemical building blocks in a report from the U.S Department of Energy (Werpy & Petersen, 2004). Five other compounds were also selected, including acetic acid, which is a common inhibitor present in biomass hydrolysates; *n*‐butanol, a potential biofuel and also widely used in the chemical industry; octanoic acid and 1,4‐butanediol, which are polyester precursors; and *p*‐coumaric acid, an aromatic polymer precursor, for which there is currently no efficient industrial production method.

*P. putida* KT2440 tolerance to these chemical compounds was tested by assaying their effect on the growth rate using different concentrations, using conditions identical to those used previously for *E. coli* K‐12 MG1655 (Rau et al., 2016). In order to compare the effect the different compounds have on *P. putida* KT2440 and *E. coli* K‐12 MG1655 growth, we calculated the concentration of each compound required to reduce the growth rate by 33%, as described previously (Rau et al., 2016). The concentrations of compounds needed to reduce the growth rate in *P. putida* KT2440 varied significantly. Some of the chemicals were found to effect growth at very low concentrations, including furfural, which was found to be toxic at a concentration of approximately 15 mM; levulinic acid and *n*‐butanol at 40 mM; and hydroxy‐*γ*‐butyrolactone at 58 mM (Table 3). Others were found to require higher concentrations to achieve the same effect on the growth, such as threonine or 1,4‐butanediol, for which concentrations of 412 and 399 mM were required, respectively.

**Table 3:** Concentrations of biochemicals leading to a 33% reduction in the growth rate (mM)

	*P. putida* KT2440	*E. coli* MG1655
Itaconic acid	189.5	215.2^*^
Succinic acid	148.3	262.5^*^
Threonine	412.0	58.8^*^
Sodium acetate	65.6	91.4^*^
Butanol	40.2	82.0^*^
Levulinic acid	40.0	67.0^*^
1,4‐butanediol	398.7	452.7^*^
Furfural	14.9	12.0^*^
3‐hydroxy‐*γ*‐butyrolactone	58.0	133.7^*^
Octanoic acid	39.5	15.0
*p*‐Coumaric acid	61.0	30.4

^*^ Data obtained from (Rau et al., 2016).

When comparing to *E. coli* K‐12 MG1655, most of the chemical compounds tested were found to have a similar or even higher inhibitory effect on *P. putida* KT2440 in the conditions used. However, higher tolerance toward L‐threonine, octanoic acid, *p*‐coumaric acid, and furfural was found in *P. putida* KT2440. The highest difference in tolerance was found for L‐threonine, where *P. putida* KT2440 was found to be sevenfold more tolerant when compared to *E. coli* K‐12 MG1655. *P. putida* KT2440 was also found to be twofold more tolerant to octanoic acid and *p*‐coumaric acid, whereas a lower difference of 1.25‐fold increased tolerance was found for furfural (Table 3). Since *p*‐coumaric acid is a known toxic compound in biomass hydrolysate, and since its toxicity has been shown to affect production by fermentation (Sariaslani, 2007), we decided to study the mechanism of tolerance toward this compound in further detail.

### 3.2 The deletion of *fcs* does not affect the tolerance toward *p*‐coumaric acid

**Figure 2:**
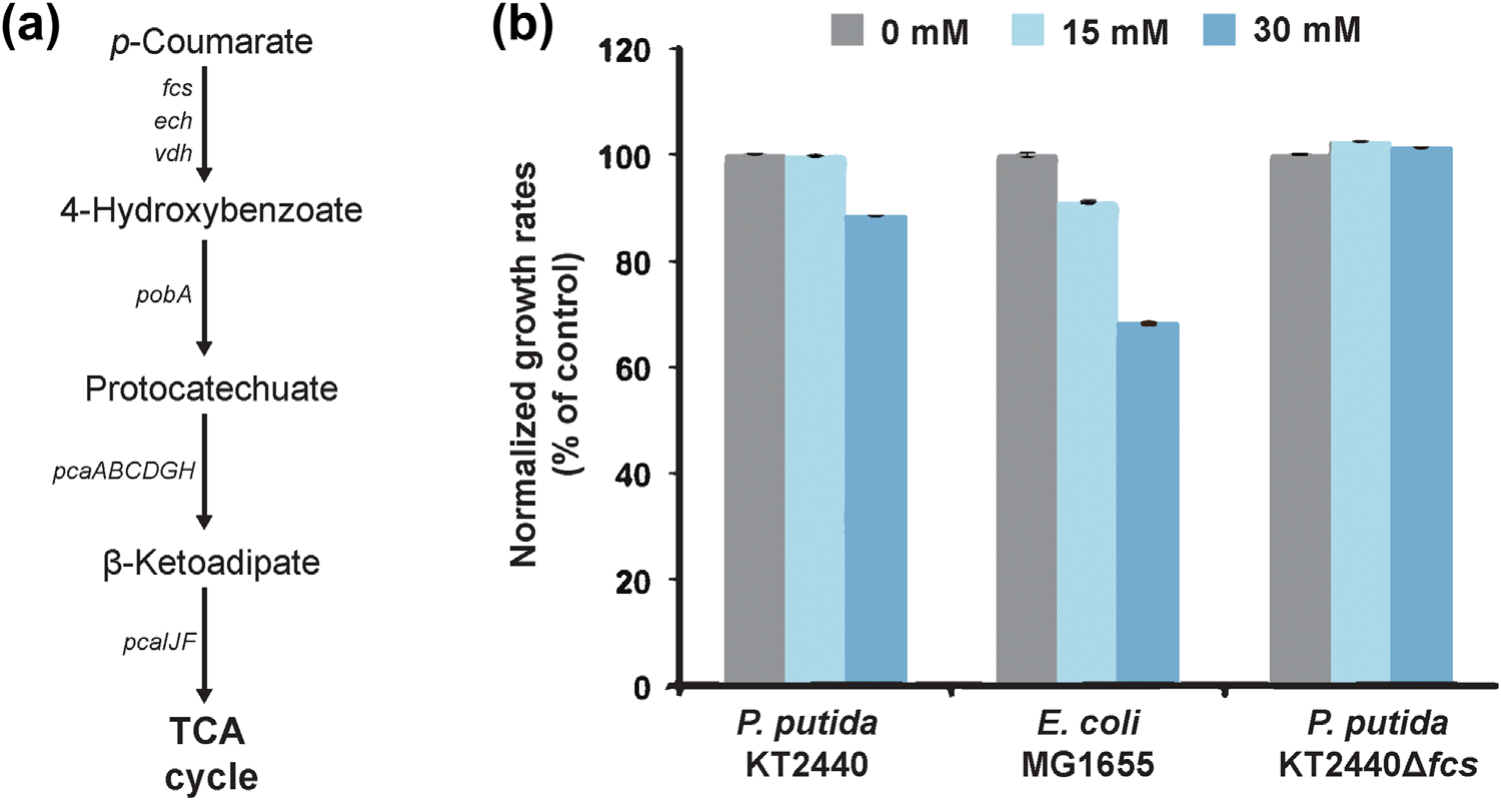
(a) Degradation pathway of *p*‐coumaric acid in *P. putida* KT2440 ending in the tricarboxylic acid (TCA) cycle. (b) Growth rates of *P. putida* KT2440, *E. coli* K‐12 MG1655, and *P. putida* KT2440 Δ*fcs* when exposed to different concentrations of *p*‐coumaric acid. Growth rates were normalized to the growth rate in control conditions without *p*‐coumaric acid. Error bars indicate the standard deviation of four different biological replicates

*P. putida* KT2440 is able to degrade *p*‐coumaric acid and use it as carbon source (Figure 2a) (Jiménez, Miñambres, Luis, & Díaz, 2002). In order to investigate the effect of the degradation of *p*‐coumaric acid on tolerance, a strain with a deletion of the first gene in the *p*‐coumaric acid degradation pathway, *fcs*, which abolishes *p*‐coumaric acid degradation, or conversion to another compound (Calero et al., 2016), was tested in different concentrations of the compound. Its tolerance to *p*‐coumaric acid was compared to the wild‐type strain, which is able to degrade *p*‐coumaric acid, and to *E. coli* K‐12 MG1655. Whereas a *p*‐coumaric acid concentration of 30 mM was enough to reduce the growth rate of *E. coli* to 68% of the control grown without the compound, no effect on growth was observed when this concentration was added to the *P. putida* KT2440 Δ*fcs* strain (Figure 2b).

### 3.3 Tn*5* library generation in *P. putida* KT2440 and *putida* KT2440 Δ*fcs*

Since degradation of *p*‐coumaric acid was shown not to be involved in the increased tolerance of *P. putida* KT2440 toward *p*‐coumaric acid, a random library of mini‐Tn*5* mutants was generated in order to further investigate the possible mechanisms of tolerance (Figure 1b). To obtain a mutant library with a high genomic coverage, a triparental mating of two plasmids, pUT‐Km, containing the transposon, and pRK600, a conjugation helper plasmid was performed (Herrero et al., 1990). To have a heterologous antibiotic resistance cassette for selection of the recipients with the mini‐Tn*5* insertions and to avoid growth of other strains used in the triparental mating, a gentamycin resistance gene was introduced into the genome of *P. putida* KT2440 and *P. putida* KT2440 Δ*fcs* using a mini‐Tn*7*‐Gm transposon (Figure 1b). This approach inserts the gene in a fixed position on the genome, after the gene *glmS*, which has previously been shown to be innocuous to the cell physiology (Lambertsen, Sternberg, & Molin, 2004). Using this method we avoided the natural chloramphenicol resistance of *P. putida* KT2440 as a selection marker, which would introduce a bias in the transposon insertion sequencing results for mutants involved in the chloramphenicol resistance, such as the efflux pump TtgABC (Fernandez, Conde et al., 2012). The use of alternative carbon sources, like citrate, that only the receptor strain could degrade was also avoided, and the developed method thereby enabled the use of LB in the creation of the library.

An efficiency of 10^−6^ CFU/conjugant cells when using triparental mating was achieved (Figure 1b). Using this method, a pooled library of ∼125.000 mutants in *P. putida* KT2440 and ∼95.000 mutants in the strain *P. putida* KT2440 Δ*fcs* was generated. The number of transposon insertions corresponds to a theoretical insertion average of one insertion every 52 bp or one insertion every 65 bp, respectively.

**Figure 3:**
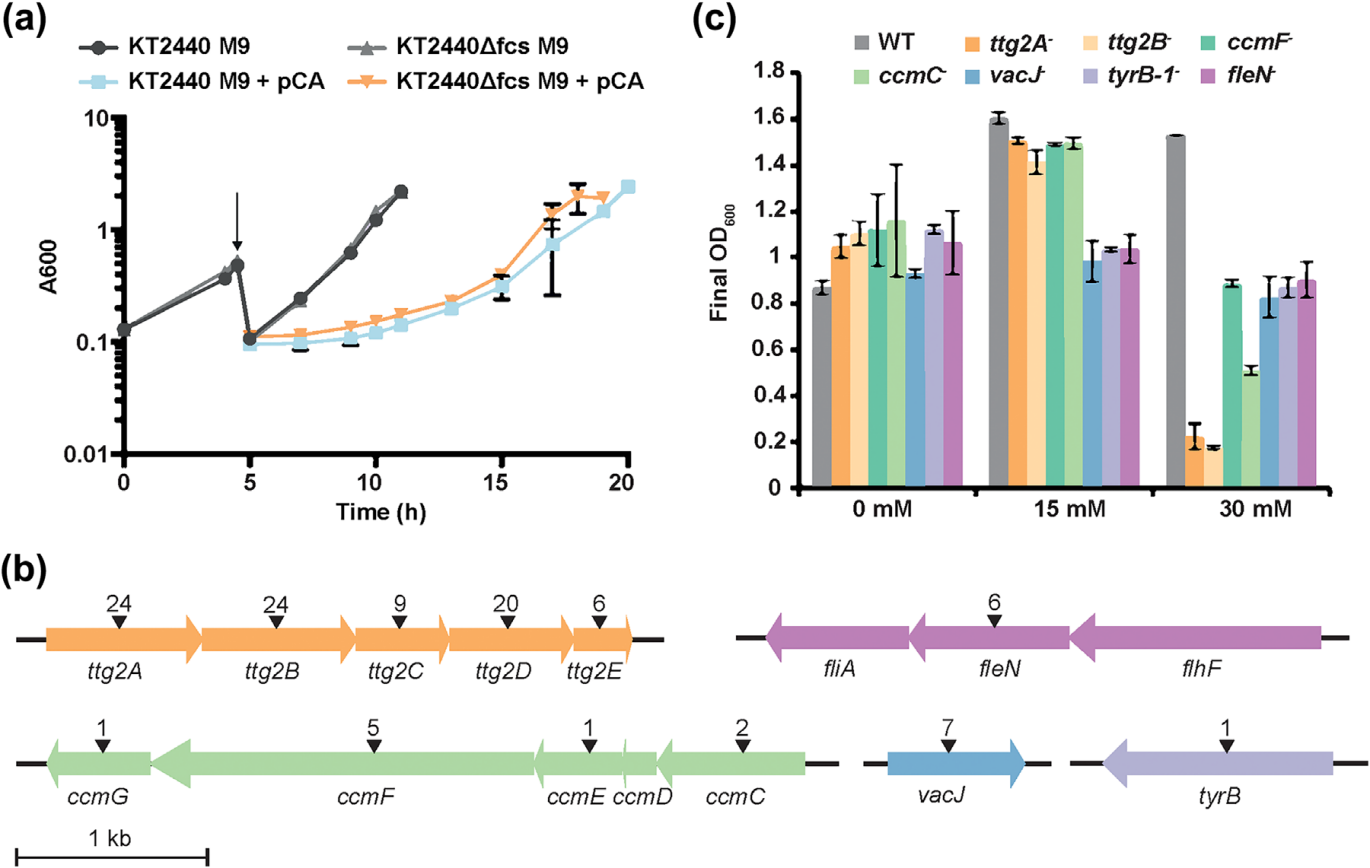
(a) Growth of the Tn5 libraries in *P. putida* KT2440 and *P. putida* KT2440 Δ*fcs* in M9 and M9 supplemented with 50 mM of *p*‐coumaric acid (pCA) is shown. The arrow indicates when *p*‐coumaric acid was added to the cultures. The growth experiments ended when cells were harvested for DNA sequencing. (b) Some of the most important genes identified to be involved in the tolerance of *P. putida* KT2440 toward *p*‐coumaric acid in their genetic landscape. The number of different insertions found in the Tn‐seq assay is shown on top of each gene with arrows. (c) Final OD_600_ of strains of *P. putida* KT2440 (WT), *ttg2A^‐^*, *ttg2B^‐^*, *ccmF^‐^*, *ccmC^‐^, vacJ^‐^, tyrB^‐^*, and *fleN^‐^* at three different concentrations of *p*‐coumaric acid: 0, 15, and 30 mM. Error bars indicate standard deviation of three independent biological replicates

The two independent *P. putida* KT2440 and *P. putida* KT2440 Δ*fcs* libraries of random transposon insertion mutants were grown in modified M9 minimal media with or without supplementation of 50 mM *p*‐coumaric acid as stressor. A comparison of the resulting populations of random insertions in each condition can be used to identify genes that are related to the growth under the stressful conditions. Cells were harvested for further analysis during late exponential growth, after approximately five generations in the presence of *p*‐coumaric acid (Figure 3a). The growth rate of the transposon insertion libraries when exposed to *p*‐coumaric acid stress was 77% and 83% for *P. putida* KT2440 and *P. putida* KT2440 Δ*fcs*, respectively, compared to the libraries that were grown in modified M9 minimal medium, corresponding to increased doubling times from 82 to 107 min in the *P. putida* KT2440 strain, and from 81 to 98 min in the *P. putida* KT2440 Δ*fcs* strain. The populations furthermore had an approximately 6 hr lag phase compared to the cultures without *p*‐coumaric acid.

### 3.4 Tn‐seq results demonstrate that the structure of the membrane has an important role in the tolerance of *putida* KT2440 to *p*‐coumaric acid

Sequencing of the junctions between the transposon and the genome was achieved by generating an enriched library as previously described (Lennen & Herrgård, 2014). A pUT‐Km transposon specific primer, BioTEG‐Tn5‐fw, was designed for the enrichment of the junctions (Table 2). Sequencing of the enriched library resulted in the identification of approximately 12.900 different insertions with 20 reads or more in the library derived from *P. putida* KT2440 Δ*fcs*, whereas approximately 6,000 insertions with 20 reads or more were found in the *P. putida* KT2440 library. Using the generated libraries, we sought to identify genes involved in tolerance toward *p*‐coumaric acid. A number of genes were found to be important for the growth of both *P. putida* KT2440 and *P. putida* KT2440 Δ*fcs* when exposed to high concentrations of *p‐*coumaric acid. Genes showing a very high impact on tolerance included the genes in the *ttg2ABCDE* operon, encoding an ABC (ATP‐binding cassette) transporter. Transposon insertions in all of the genes in this operon had a strong effect on growth in the presence of *p*‐coumaric acid, and these insertions were represented between 11‐ and 15‐fold less in the presence of *p*‐coumaric acid. Moreover, all the genes were found to have more than one insertion in different positions of the ORFs. The longest genes in the operon, *ttg2A*, *ttg2B*, and *ttg2D* had 16, 17, and 13 different insertion sites, respectively, in *P. putida* KT2440, and 8, 7, and 7 in strain *P. putida* KT2440 Δ*fcs*. On the other hand, *ttg2C* and *ttg2E*, which are shorter genes, were found to have 6 and 5 different insertion sites in strain *P. putida* KT2440 and 3 and 1 in strain *P. putida* KT2440 Δ*fcs*. The loss of function of two other genes, *vacJ* and *fleN*, encoding an outer membrane protein and a flagellar number regulator, respectively, was also shown to impair growth in both strains in the presence of *p*‐coumaric acid. Transposon insertions in *vacJ* were found to be approximately 15‐fold less abundant when *p*‐coumaric acid was present in the media, and mutations in *fleN* were shown to be around 15‐fold less represented when *p*‐coumaric acid was present. The gene *fleN* forms an operon together with *fliA*, *flhN*, and *flhA*, but none of the other operon members appeared to play a role in the tolerance to *p*‐coumaric acid (Table 4).

**Table 4:** Genes involved in tolerance toward *p*‐coumaric acid

Gene	Number of insertions	Fold change	Gene name	Gene function
a				
PP_0958	24	11.3	*ttg2A*	Toluene tolerance ABC efflux transporter ATP‐binding protein
PP_0959	24	10.4	*ttg2B*	Hypothetical protein
PP_0960	9	15.2	*ttg2C*	Hypothetical protein
PP_0961	20	11.7	*ttg2D*	Toluene tolerance family protein
PP_0962	6	11.1	*ttg2E*	Toluene‐tolerance protein
PP_2163	7	13.4	*vacJ*	VacJ family lipoprotein
PP_4342	6	8.16	*fleN*	Flagellar number regulator FleN
b				
PP_3286	1	9.3	*phaN*	PaaX family transcriptional regulator
PP_4320	1	8.1	*ccmH*	Hypothetical protein
PP_4321	1	7.7	*ccmG*	Thiol‐disulfide oxidoreductase
PP_4322	5	7.4	*ccmF*	Cytochrome C biogenesis protein CcmF
PP_4323	1	8.2	*ccmE*	Cytochrome C biogenesis protein CcmE
PP_4325	2	7.2	*ccmC*	Heme exporter protein CcmC
PP_4937	1	7.1	–	Toluene tolerance protein
c				
PP_0168	1	18.8	–	Surface adhesion protein
PP_0674	1	6.1	–	ABC transporter ATP‐binding protein
PP_1185	1	7.5	*oprH*	Outer membrane protein H1
PP_1972	1	6.3	*tyrB‐1*	Aromatic amino acid aminotransferase
PP_2889	2	10.2	–	Transmembrane anti‐sigma factor

a, Important genes found in both strains; b, Important genes found in the strain *P. putida* KT2440 *fcs*; c, Important genes found in the strain *P. putida* KT2440.

A number of transposon insertions were also identified in just one of the two strain backgrounds. One of the most significant was the operon consisting of the genes *ccmCDEF* and *dsbE* in the strain *P. putida* KT2440 Δ*fcs*. All the genes in this operon, which encodes the cytochrome *c* maturation system, were shown to be involved in tolerance toward *p*‐coumaric acid, except for *ccmD*, likely due to the fact that it is the shortest gene in the operon, with approximately 200 bp and therefore may not have been targeted by transposon insertions. The genes in the operon with intermediate lengths (450–550 bp), *dsbE* and *ccmE* were targeted by one type of insertion whereas the longest genes, *ccmF* and *ccmC* (2,000 and 750 bp, respectively) were found to have five and two different insertions, respectively. In all cases, the mutants in these genes were found to be represented approximately 13‐fold less in the populations when *p*‐coumaric acid was present in the media. In the *P. putida* KT2440 library, genes encoding some other membrane proteins such as the outer membrane protein OprH, a surface adhesion protein encoded by gene PP_0168 and an ABC transporter encoded by PP_0674 were found to be related to the tolerance toward *p*‐coumaric acid (Table 4).

The loss‐of‐function of two genes was found to increase the growth rate of both *P. putida* KT2440 Δ*fcs* and *P. putida* KT2440 in the presence of *p*‐coumaric acid. These genes include the LysR family transcriptional regulator encoded by the gene PP_1262 and the MarR family transcriptional regulator encoded by the gene PP_4515. In both cases, the cells containing transposon insertions in these genes were found to grow better when *p*‐coumaric acid was present and were approximately 15‐fold more abundant under these conditions.

### 3.5 Loss of function in genes identified through the Tn‐seq leads to impaired growth in the presence of *p*‐coumaric acid

Two complete operons, *ttg2* and *ccm*, were identified in both the assayed strain as playing an important role in the tolerance of *P. putida* KT2440 toward *p*‐coumaric acid (Figure 3b). To further investigate the role of these genes, independent insertion mutants available from the PRCC (Duque et al., 2007) in genes *ttg2A*, *ttg2B*, *ccmC*, and *ccmF* were acquired, while insertion mutants in genes *vacJ*, *tyrB*, and *fleN* were constructed. The different strains were grown in medium containing different concentrations of *p*‐coumaric acid, and a clear impact on the final optical density of the mutant strains was found when compared to the wild‐type strain (Figure 3c), and a similar effect was observed for the growth rate (Figure S1). This result confirms that both the *ttg2* and *ccm* operons as well as other genes identified through the Tn‐seq experiment are strongly involved in the tolerance toward the aromatic compound *p*‐coumaric acid.

## 4 DISCUSSION

When compared to *E. coli*, *P. putida* KT2440 was found to have enhanced tolerance to only three out of the 11 tested industrially relevant compounds, when these were supplemented to the growth medium. Even though *P. putida* strains have been extensively studied for their tolerance to certain organic solvents such as toluene, *P. putida* KT2440 was not found to be more tolerant toward carboxylic acids (levulinic acid, acetic acid, itaconic acid, and succinic acid) and furfural when compared to *E. coli*. Surprisingly, *P. putida* KT2440 was not found to be more tolerant toward *n*‐butanol, even though some *P. putida* strains, including *P. putida* DOT‐T1E and S12, have a high tolerance toward organic solvents such as *n*‐butanol (Rühl, Schmid, & Blank, 2009). It should be noted that the compounds were added to the growth medium in the given experiments. It is possible that the effect and tolerance mechanisms could be different if the compounds were produced intracellularly.

*P. putida* KT2440 was shown to be significantly more tolerant toward *p*‐coumaric acid when compared to *E. coli*. Deletion of the first gene (*fcs*) of the degradation pathway revealed that degradation of the compound was not the mechanism of detoxification. This observation agrees with previous observations showing that even though metabolism of toxic chemicals can help to alleviate toxicity, it is usually a minor mechanism of tolerance (Ramos et al., 2002). Interestingly, some defect on the growth was observed only for the wild‐type strain when exposed to 30 mM of *p*‐coumaric acid, and not for the deletion mutant, suggesting that certain intermediates in the degradation pathway may be toxic (Figure 2b).

To further investigate the mechanism of tolerance toward *p*‐coumaric acid, we developed a new method for generating large transposon libraries compatible with Tn‐seq. By comparing sequences of libraries grown with or without inhibitory concentrations of *p*‐coumaric acid, we identified 19 genes involved in the tolerance to *p*‐coumaric acid, many of them involved in membrane processes. These results are in accordance to a previous study that investigated the mechanism of action of *p*‐coumaric acid in different bacteria, and pointed toward membrane disruption as a main effect (Lou et al., 2012). The genes in the ABC (ATP‐binding cassette) transporter, Ttg2ABC, were part of the seven genes for which loss‐of‐function indicated impaired growth in both strain libraries when exposed to high concentrations of *p*‐coumaric acid. This transporter has previously been identified to play a role in the tolerance toward toluene in *P. putida* (Garcia et al., 2010; Kim, Lee, Lee, & Lim, 1998), and it has been described to be involved in resistance to a number of other stress agents (Garcia et al., 2010).

Another gene with decreased abundance in both libraries when exposed to *p*‐coumaric acid, *vacJ* (PP_2163), contains the domain of the VacJ superfamilies. VacJ has been characterized as an outer membrane protein associated to an ABC transporter system in other organisms, such as *E. coli* and *Shigella flexneri*, in which it has been proposed to be involved in the maintenance of outer membrane stability in the presence of membrane disruptors such as SDS (Carpenter et al., 2014; Malinverni & Silhavy, 2009). Its deletion has been shown to increase permeability of the outer membrane (Malinverni & Silhavy, 2009) and increase the formation of vesicles in *E. coli* and other gram‐negative bacteria (Roier et al., 2016). In *P. aeruginosa*, a VacJ homolog has been described to play a role in antibiotic resistance. VacJ has also been shown to be more abundant in cells growing in the presence of phenol in *P. putida* KT2440 (Santos et al., 2004).

A transposon insertion in the flagellar number regulator *fleN* was shown to be important for tolerance toward *p*‐coumaric acid. The deletion of this gene in *P. aeruginosa* has been shown to alter the single polar flagella localization, affecting the motility of the cells (Dasgupta, Arora, & Ramphal, 2000). Even though some genes of the flagella systems have been identified in the tolerance toward toluene in the strain *P. putida* DOT‐T1E, their function in the resistance is not clear, since it does not seem to be related to the cells motility (Ramos et al., 2002).

In one of the libraries, the complete *ccmCDEF‐dsbE* operon, containing the genes PP_4321‐PP_4325, was found to be of especial importance for tolerance toward *p*‐coumaric acid. These genes encode the cytochrome *c* maturation system, which is known to be present in Proteobacteria, where it promotes the attachment of the heme group to the apocytochrome *c* in the periplasm, allowing the correct function of the type *c*‐cytochrome in cell respiration and ATP synthesis (Thöny‐Meyer, 2000). The effect of the lack of a functional operon in tolerance toward *p*‐coumaric acid may indicate a high energy requirement or disruption of the membrane potential, causing the release of cytochrome *c*, as it has previously been shown in mitochondria (Shailasree, Venkataramana, Niranjana, & Prakash, 2014). Moreover, an increased relative abundance of genes involved in energy metabolism in *E. coli* has also been shown to play a role in free fatty acids stress conditions (Lennen et al., 2011). Furthermore, the cytochrome *c* maturation system has been described to play a role in other cellular processes in other organisms (Cianciotto, Cornelis, & Baysse, 2005), such as *P. aeruginosa* (Baert, Baysse, Matthijs, & Cornelis, 2008) or *P. fluorescens* (Yang, Azad, & Cooksey, 1996). A transposon insertion mutant in this operon in the strain *P. putida* P8 was found to have reduced *cis*‐*trans* isomerization of unsaturated fatty acids, since a cytochrome *c*‐type heme‐binding motif was found to catalyze the activity of this enzyme.

In addition to the genes discussed above, other genes such as the transcriptional repressor *phaN*, a number of outer membrane proteins and surface adhesion proteins as well as other ABC transporters (PP_0168, PP_0674, and PP_1185) were identified to be important for tolerance, most of them in the *P. putida* KT2440 background. It is possible that these genes may be related to the detoxification of the intermediates of the *p*‐coumaric acid degradation pathway. The identification of these efflux pumps supports the observation that the role of transporters are of major importance when *P. putida* KT2440 is exposed to chemical stress, as it has also been observed in other organisms such as *Clostridium acetobutylicum* in the presence of butanol.

The importance of some of these genes was validated by assessing the effect of *p*‐coumaric acid on the growth of a number of independent insertion mutants in the genes *ttg2A* and *ttg2B*, *ccmC* and *ccmF*, *vacJ*, *tyrB*, and *fleN*, which displayed clear growth impairment at high concentrations of *p*‐coumaric acid when compared to the wild‐type (Figure 3b).

One of the beneficial insertions found, in gene PP_4515, is annotated in *P. putida* KT2440 as belonging to the MarR transcriptional regulator family. However, a BLASTp analysis of the protein sequence showed that this transcriptional regulator seems to be more closely related to the transcriptional regulator SlyA, with a 38% of identity in the amino acid sequence. SlyA belongs to a large family of regulators involved in different processes, and it has been shown to regulate chaperones and other stress proteins in one strain of *E. coli* (Spory et al., 2002). However, the activity of various homologs differs widely among species, thus further work is required in order to understand the cellular mechanism in *P. putida* KT2440 of this regulator. The other gene, PP_1262, belonging to the LysR‐type transcriptional regulator family has no known function. The LysR‐type transcriptional regulator family has been described to regulate a number of functions, such as aromatic acid metabolism, and both activators and repressors have been identified. Most of them are described to be transcribed divergently of the genes they regulate. Here, the operon PP_1263 to PP_1266, which is divergently transcribed, has been annotated as an efflux system for the export of fusaric acid, a fungal toxin released by phytopathogenes (Martins Dos Santos, Heim, Moore, Strätz, & Timmis, 2004). Further studies are required to elucidate the effect of this regulator and the genes that it possibly regulates for the tolerance.

In conclusion, we have developed a method for generating large libraries of random insertion mutants in *P. putida* KT2440 with a minimum amount of bias by avoiding natural *P. putida* selection methods. *P. putida* KT2440 was found to have enhanced tolerance toward *p*‐coumaric acid when compared to *E. coli*. Using Tn‐seq analysis, the mechanisms of tolerance were found to involve membrane stability, exclusion, and secretion of the toxic compound to the outside of the cell. Due to its significant tolerance, *P. putida* could be a promising production host for p‐coumaric acid. The developed Tn‐seq method will be generally useful for investigating a wide range of conditions in this organism.

## CONFLICTS OF INTEREST

The authors declare no conflict of interest.

## Supporting information

Additional Supporting Information may be found online in the supporting information tab for this article.

**Figure S1**. Growth curves of the *P. putida* KT2440 (WT) in M9 (blue) and M9 supplemented with *p*‐coumaric acid at a concentration of 15 (red) and 30 (green) mM in 96‐well microtiter plates.
