# Supplementary material for: Genome‐wide identification of tolerance mechanisms toward p‐coumaric acid in Pseudomonas putida
Source: Biotechnol Bioeng. 2017 Nov 28;115(3):762–74. doi: 10.1002/bit.26495 (PMC5814926; doi:10.1002/bit.26495)
Supplement: Supplementary file 1 — Figure S1. Growth curves of the P. putida KT2440 (WT) in M9 (blue) and M9 supplemented with p‐coumaric acid at a concentration of 15 (red) and 30 (green) mM in 96‐well microtiter plates. [file BIT-115-762-s001.docx]

**Genome-wide identification of tolerance mechanisms towards *p*-coumaric acid in *Pseudomonas putida***

**RUNNING TITLE:** Genome-wide tolerance mechanisms in *P. putida*

**AUTHORS:** Patricia Calero, Sheila I. Jensen, Klara Bojanovič, Rebecca Lennen, Anna Koza, Alex T. Nielsen*

Novo Nordisk Foundation Center for Biosustainability, Technical University of Denmark, Building 220, Kemitorvet, 2800 Kgs. Lyngby, Denmark

***Corresponding author:** Alex T. Nielsen: [atn@biosustain.dtu.dk](mailto:atn@biosustain.dtu.dk)

**Figure S1:** Growth curves of the *P. putida* KT2440 (WT) in M9 (blue) and M9 supplemented with *p*-coumaric acid at a concentration of 15 (red) and 30 (green) mM in 96-well microtiter plates.
